# The Veterinary Association of Manitoba

**Published:** 1900-03

**Authors:** F. Torrance

**Affiliations:** Secretary


Secretary.

### THE VETERINARY ASSOCIATION OF MANITOBA.

THE annual meeting of this Association was held in the city of Winnipeg, February 20, 1900, the President, Dr. H. D. Smith, in the chair. A large and representative gathering of members from all parts of the province made this one of the best meetings the Association has ever held. The auditors reported the financial affairs in first-rate condition, and the Secretary showed that the membership now amounted to sixty-eight—the largest in the history of the Association.

Several interesting papers were read. Mr. W. Swenerton, of Carberry, reported a case of "Equine Tuberculosis" which presented many interesting features. In the discussion which followed Dr. Martin stated the facts relating to a similar case in his own practice, where the evidence of infection from tuberculous cows was very strong, the horse in question belonging to a dairyman who stabled the animal with his cows.

Mr. J. A. Stevenson then read a most interesting essay on "Hog Cholera"—a disease which is, fortunately, almost unknown in this province. The last outbreak was about the year 1884, and up to the present the province was free from it. Owing to stringent measures now in force the present outbreak was confined to a few farms in the vicinity of Carman, and he had every reason to think it would be successfully stamped out.

This was followed by an essay on "Tuberculosis," by Mr. W. H. Lake, of Miami, which, in the absence of the essayist, was read by the Secretary.

A motion of condolence upon the death of our late Vice-President, J.

Spiers, of Virden, was passed and the Secretary instructed to forward a copy to Mrs. Spiers.

The following gentlemen were elected officers for the ensuing year: President, J. G. Rutherford, M. P., of Portage la Prairie; Vice-President, J. F. Fisher, V.S., Brandon; Secretary-Treasurer and Registrar, F. Torrance, D.V.S., Winnipeg; Councillors, W. A. Dunbar, V.S.; W. R. Taylor, V.S., W. E. Martin, V.S., and A. E. Williamson, V.S. The examiners are Messrs. Dunbar, Martin, and Torrance.

F. Torrance,
Secretary.

